# Pre-Treatment and Post-Treatment Demodex Densities in Patients under Immunosuppressive Treatments

**DOI:** 10.3390/medicina56030107

**Published:** 2020-03-03

**Authors:** Hacer Keles, Esra Pancar Yuksel, Fatma Aydin, Nilgun Senturk

**Affiliations:** Dermatology Department, Faculty of Medicine, Ondokuz Mayıs University, Samsun 55139, Turkey; dr.hacer_keles@hotmail.com (H.K.); bennet@mynet.com (F.A.); nilsenturk@yahoo.com (N.S.)

**Keywords:** azathioprine, corticosteroids, cyclosporine, demodex, immunosuppressive treatment, methotrexate

## Abstract

*Background and Objectives*: Demodex species are common obligatory parasites and normally present in low number in human beings. Immunosuppression was suggested to be associated with increased density of Demodex mites. Systemic glucocorticoids, cyclosporine, methotrexate, and azathioprine are commonly used immunosuppressive agents. We aim to determine the pre- and post-treatment Demodex densities in patients receiving immunosuppressive therapy and compare with those of healthy subjects. *Materials and Methods*: Demodex density was investigated at the beginning, first, and third months of the immunosuppressive therapy in 45 patients who received methotrexate, cyclosporine, systemic steroid, or azathioprine treatments and in 45 healthy subjects at the same time as the patients. Five standardized skin surface biopsies were taken from cheeks, forehead, nose, and chin of the patients and control group. The presence of five or more parasites in 1 cm^2^ area was considered as positive. *Results*: Demodex test was negative at the beginning of the treatment in all patients. Demodex test was positive in one patient in the first and third months of treatment and in three patients only in the third month of treatment. In the control group, Demodex test was determined as positive in just one healthy individual at the beginning, first and third months of the study. When the patient and control groups were evaluated in terms of Demodex number, there was a statistically significant difference in Demodex density in patients treated with immunosuppressive treatment in the first and third months when compared with the control group (*p* < 0.05). *Conclusion*: Immunosuppressive treatment might increase the number of Demodex mites and demodicidosis should be kept in mind in patients on immunosuppressive treatment.

## 1. Introduction 

The two Demodex species *Demodex folliculorum* and *Demodex brevis* are common obligatory parasites of the pilosebaceous units in human beings. *Demodex folliculorum* is usually found in the infundibulum of hair follicle and *Demodex brevis* in the sebaceous glands and ducts [1,2]. The two Demodex species were reported to be present worldwide with an incidence ranging from 25% to 93% [3]. They can appear anywhere on the face but mainly on forehead, cheeks, nose, nasolabial folds and, normally present less than 5 parasites/cm^2^. But when their number increases, they might be the cause of dermatoses with chronic inflammatory eruptions [4,5,6]. Rosacea is the prominent dermatological disorder in which an association was defined between disease development and infestation with Demodex mites. *Demodex folliculorum* density was reported to be increased in patients with rosacea [7,8].

Immunosuppression was suggested as one of the causes of increased density of Demodex mites. Tacrolimus ointment was thought to be the possible cause of overgrowth of *Demodex folliculorum* because of the immunosuppressive properties. Immunocompromised patients with acquired immunodeficiency syndrome developed facial lesions of demodicidosis. Leukemia and lymphoma patients receiving chemotherapy were also found to have increased number of mites [9,10,11,12]. Since immunosuppressive agents are also frequently used for inflammatory or autoimmune skin diseases, we aimed to determine the number of Demodex mites before and after the immunosuppressive treatment in patients with dermatological disorders in this study. 

## 2. Materials and Methods

### 2.1. Patients Examined

This study included 45 patients (21 male, 24 female; mean age 42.6 ± 16 years) who were admitted to the Dermatology Clinic and were treated with immunosuppressive therapies for psoriasis vulgaris, pemphigus vulgaris, alopecia areata, or lichen planus. Patients with facial lesions were excluded from the study. The control group consisted of 45 healthy subjects (17 male, 28 female; mean age 34.6 ± 15 years). The study protocol was approved by the Ondokuz Mayıs University, Clinical Research Ethics Committee with code number OMU-KAEK-2014-805 on 25.09.2014.

### 2.2. Testing for Demodex

Five standardized skin surface biopsies were taken from the cheeks, forehead, nose, and chin of the patients at the beginning, first, and third months of the immunosuppressive treatment. Five standardized skin surface biopsies were also taken from the same regions of control subjects at the same time with the patients. For standardized skin surface biopsy, a drop of cyanoacrylate adhesive was put on 1 cm^2^ pen-marked area of a microscope slide. The adhesive-bearing surface of the slide is pressed over the skin for about 30 s. Then the slide was gently detached from the skin, clarified with 2–3 drops of glycerin, and covered with a coverslip. The slides were assessed for parasites by light microscope at magnifications of ×10. Determination of five or more parasites in 1 cm^2^ area was considered as positive [13]. 

### 2.3. Statistical Analysis

All statistical analyses were performed using the SPSS version 15 (SPSS Inc, Chicago, IL, USA). The Kolmogorov-Smirnov/Shapiro–Wilk tests were used to analyze the normal distribution. Frequency tables were used for ordinal variables and they were evaluated by chi-square test, Mann–Whitney U-test, Friedman test, Wilcoxon test. A *p*-value < 0.05 was considered statistically significant.

## 3. Results

Of all the patients 29 (64%) were psoriasis vulgaris, 7 (16%) were pemphigus vulgaris, 5 (11%) were alopecia areata, and 4 (9%) were lichen planus. When the patients were examined in terms of treatment, fifteen patients were treated with cyclosporine, fifteen patients were treated with methotrexate, nine patients were treated with only corticosteroids, and six patients were treated with both corticosteroids and azathioprine.

Demodex test was negative at the beginning in all patients. However, Demodex test was positive in one patient in the first and third months of treatment and in three patients in the third month of treatment. The patient whose Demodex test was positive at the first and third months was a female pemphigus vulgaris patient and treated with systemic steroid and azathioprine. Two of the patients, whose Demodex tests were negative at the first month but positive at the third month, were also female pemphigus vulgaris patients under systemic steroid and azathioprine. The other patient, whose Demodex test was negative at the first month but positive at the third month, was a male psoriasis vulgaris patient receiving methotrexate.

In the control group, Demodex test was determined as positive in only one healthy individual at the beginning, first and third months of the study. The rest of the individuals in the control group had negative Demodex test at the beginning, first and third months.

Considering the number of mites, Demodex mites were observed in 16 patients at the beginning, in 28 patients in the first month and in 27 patients in the third month. 

The number of healthy individuals in which Demodex mites observed was 12 at the beginning, 11 at the first month, and 14 at the third month of the study in the control group. Demodex densities of patient and control groups were presented in Table 1.

When the patient and control groups were evaluated in terms of Demodex density, no statistically significant difference was found in the Demodex density at the beginning of the treatment. On the other hand there was a statistically significant difference in Demodex density in patients treated with immunosuppressive therapy in the first and third months when compared with the control group (*p* < 0.05).

## 4. Discussion

Although Demodex mites might be seen in healthy people, density remains low. They are acquired shortly after birth by the mother to infant transmission and increase in number with sebaceous gland proliferation during puberty. They become pathogenic when they multiply and reach to the density of >5 mites/cm^2^ of skin [2,6]. However factors affecting the density of Demodex mites are still not known exactly. Some local or systemic factors may induce the proliferation of mites. Immune deficiency was suggested to be the possible cause of overgrowth of mites [10,11]. 

Demodicidosis was observed in immunocompromised patients. It was described as an opportunistic infection of the skin in immunocompromised children who were receiving chemotherapy with the diagnosis of acute lymphoblastic leukemia. A pruritic, papulopustular facial eruption was described in those patients [12,13,14,15]. Similarly demodicidosis was also described in adult leukemia and lymphoma patients. The acute myelocytic leukemia patients receiving treatment of cytosine arabinoside, daunorubicin, hydroxyurea, and mitozantrone were reported with the highest Demodex density. Demodicidosis was suggested in the differential diagnosis of facial eruptions in patients with hematological malignancies on chemotherapy [6]. Another group of immunocompromised patients who were also reported to be predisposed to Demodicidosis was patients with acquired immunodeficiency syndrome. Erythematous papulopustules were described in those patients but just one had an unusual manifestation of Demodex infestation with ivory-white, poorly defined, indurated area. Alterations in humoral and cell-mediated immunity may allow the proliferation of Demodex and so the pathogenic role of it [16,17,18]. 

Patients with chronic renal failure were reported to have defective host defenses [19]. Uremia causes alterations in different aspects of immune response such as impaired oxidative response of neutrophils and abnormal function of lymphocytes [19]. Therefore, proliferation of the normally commensal mites might be allowed by host immune dysfunction. This was shown in the study investigating the incidence of *Demodex folliculorum* in end stage renal failure patients, the number of mite was found to be increased and the mean number of *Demodex folliculorum* was reported as 6.12/cm^2^ in those patients. Presence of more than five Demodex in a 1 cm^2^ area was found in 27 patients among 67 end stage renal failure patients. [20]. Similarly Duzgun et al. [21] also reported higher mean mite density in hemodialysis patients than controls. 

Tacrolimus an immunosuppressive macrolide inhibits cytokine transcription and activation of T cells [9]. Rosaceiform dermatitis as a complication of treatment with tacrolimus ointment was reported. Increased Demodex mites determined by biopsy in two of the patients treated with tacrolimus ointment and proliferation of Demodex was suggested as the result of local immunosuppression caused by topical tacrolimus treatment [22]. Rosaceiform dermatitis with follicular Demodex was also reported by Lübbe et al. [23] with pimecrolimus 1% cream. Malignancy and the malnutrition were the other known immune deficiency states. Both conditions were found to be associated with increased Demodex [24]. In the study analyzing the frequency of *Demodex folliculorum* infestation in patients with urological cancers, possible relationship between the cancer as the immunosuppressive state, and the increased *Demodex folliculorum* incidence was detected [25]. Demodex mites were also reported to be isolated from eyelashes and associated with blepharitis [26,27,28].

Some systemic agents have the property of causing immune dysregulation. Systemic glucocorticoids increase susceptibility to many bacterial, viral, fungal, and parasitic infections while reducing the activation and proliferation of effector T cells and also B cell function [29]. Cyclosporine reduces CD4+ and CD8+ T cells in the epidermis, methotrexate inhibits DNA synthesis in immunologically active cells and causes pancytopenia, and azathioprine suppresses T-cell function, B-cell antibody production, and ability of Langerhans cells to present antigens [30]. In this study, we investigated the density of Demodex in patients receiving these agents. The combination treatment such as systemic glucocorticoid and azathioprine caused the prominent increase in Demodex density in our study compared to monotherapy. 

The results of our study suggest that there is an association between immunosuppressive treatment and the number of Demodex mites. Patients receiving immunosuppressive therapy showed statistically significant difference in Demodex density when compared with the control group. These results are consistent with the literature. However we did not find any facial eruption in our patients. We evaluated the patients just one and three months after the initiation of the treatment. This duration may not be enough to increase mites further. So if the patients had been evaluated after a longer period of time, greater number of Demodex and provoked abnormal immunologic reaction of the skin to the parasites with the appearance of cutaneous lesions might be observed. 

## 5. Conclusions

Demodex density was higher than the control group at the first and third months of the immunosuppressive therapy and this difference was statistically significant. So, immunologic deficiency might increase the number of mites and demodicidosis should be kept in mind in patients on immunosuppressive treatment.

## Figures and Tables

**Table 1:** Demodex densities of patient and control groups at the initiation, first, and third months of therapy.

	Initiation of Treatment		First Month of Treatment		Third Month of Treatment	
	Mean Demodex Density	Minimum, Maximum Demodex Number	Mean Demodex Density	Minimum, Maximum Demodex Number	Mean Demodex Density	Minimum, Maximum Demodex Number
Disease group	0.44 ± 0.65	0/2	1.15 ± 1.39	0/8	1.84 ± 2.29	0/10
Control group	0.48 ± 1.05	0/6	0.51 ± 1.1	0/6	0.71 ± 1.39	0/8
